# Current medical research funding and frameworks are insufficient to address the health risks of global environmental change

**DOI:** 10.1186/s12940-016-0183-3

**Published:** 2016-11-11

**Authors:** Kristie L. Ebi, Jan C. Semenza, Joacim Rocklöv

**Affiliations:** 1 Department of Global Health, University of Washington, Seattle, WA 98195 USA; 2 Stockholm Environmental Institute, Linnégatan 87D, 115 23 Stockholm, Sweden; 3 Department of Public Health and Clinical Medicine, Umea University, Umea, Sweden

**Keywords:** Climate change, Global change, Human health, Funding

## Abstract

**Background:**

Three major international agreements signed in 2015 are key milestones for transitioning to more sustainable and resilient societies: the UN 2030 Agenda for Sustainable Development; the Sendai Framework for Disaster Risk Reduction; and the Paris Agreement under the United Nations Framework Convention on Climate Change. Together, these agreements underscore the critical importance of understanding and managing the health risks of global changes, to ensure continued population health improvements in the face of significant social and environmental change over this century.

**Body:**

Funding priorities of major health institutions and organizations in the U.S. and Europe do not match research investments with needs to inform implementation of these international agreements. In the U.S., the National Institutes of Health commit 0.025 % of their annual research budget to climate change and health. The European Union Seventh Framework Programme committed 0.08 % of the total budget to climate change and health; the amount committed under Horizon 2020 was 0.04 % of the budget. Two issues apparently contributing to this mismatch are viewing climate change primarily as an environmental problem, and therefore the responsibility of other research streams; and narrowly framing research into managing the health risks of climate variability and change from the perspective of medicine and traditional public health. This reductionist, top-down perspective focuses on proximate, individual level risk factors. While highly successful in reducing disease burdens, this framing is insufficient to protect health and well-being over a century that will be characterized by profound social and environmental changes.

**Conclusions:**

International commitments in 2015 underscored the significant challenges societies will face this century from climate change and other global changes. However, the low priority placed on understanding and managing the associated health risks by national and international research institutions and organizations leaves populations poorly prepared to cope with changing health burdens. Risk-centered, systems approaches can facilitate understanding of the complex interactions and dependencies across environmental, social, and human systems. This understanding is needed to formulate effective interventions targeting socio-environmental factors that are as important for determining health burdens as are individual risk factors.

## Background

2015 saw major national commitments to transition to more climate-resilient and sustainable societies, in part by preparing for and managing the challenges and opportunities of global environmental change (Table 1). The United Nations launched the 2030 Agenda for Sustainable Development that calls on countries to begin efforts to achieve 17 Sustainable Development Goals (and 169 targets) over the next 15 years. The goals, unanimously adopted by the UN’s 193 Member States in September, address the social, economic, and environmental dimensions of sustainable development, as well as promoting peace, justice, and effective institutions [1]. Goal 3 aims to improve population health (good health and well-being), with health embedded in multiple other goals, including no poverty, zero hunger, clean water and sanitation, gender equality, reduced inequalities, sustainable cities. Therefore, health is central to transitioning to more sustainable and resilient pathways. Two other commitments in 2015 of importance for environmental health were the Sendai Framework for Disaster Risk Reduction 2015–2030 (adopted in March 2015); and the Paris Agreement under the United Nations Framework Convention on Climate Change (agreed in December 2015). On 5 October 2016, the threshold for entry into force of the Paris Agreement was achieved; the Agreement will enter into force on 4 November 2016. Further, in October 2016, 170 countries signed a legally binding accord to limit climate change by limiting the worldwide use of chemical coolants called hydrofluorocarbons (HFCs) used in air-conditioners and refrigerators.

**Table 1:** Major international commitments in 2015 relative to human health and well-being

Sustainable development goals	Sendai framework for disaster risk reduction	Paris agreement (United Nations Framework Convention on Climate Change
• No poverty • Zero hunger • Good health and well-being • Quality education • Gender equality • Clean water and sanitation • Affordable and clean energy • Decent work and economic growth • Industry, innovation, and infrastructure • Reduced inequalities • Sustainable cities and communities • Responsible consumption and production • Climate action • Life below water • Life on land • Peace and justice, strong institutions • Partnerships for the goals	• Understanding disaster risk • Strengthening disaster risk governance to manage disaster risk • Investing in disaster risk reduction for resilience • Enhancing disaster preparedness for effective response and to “build back better” in recovery, rehabilitations, and reconstruction	This Agreement, in enhancing the implementation of the Convention, including its objective, aims to strengthen the global response to the threat of climate change, in the context of sustainable development and efforts to eradicate poverty, including by: • Holding the increase in the global average temperature to well below 2 °C above pre-industrial levels and to pursue efforts to limit the temperature increase to 1.5 °C above pre-industrial levels, recognizing that this would significantly reduce the risks and impacts of climate change; • Increasing the ability to adapt to the adverse impacts of climate change and foster climate resilience and low greenhouse gas emissions development, in a manner that does not threaten food production; • Making finance flows consistent with a pathway towards low greenhouse gas emissions and climate resilient development.

The 2030 Agenda for Sustainable Development recognizes that disaster risk management is an integral part of social and economic development (Goal 11). Disaster risk management has shifted from a hazard and response-driven approach to a risk-driven, integrated culture that considers prevention, recovery, and rehabilitation [2]. The Framework promotes implementation in coordination with other frameworks, such as the International Health Regulations. With climate change projected to increase the frequency and intensity of many extreme weather and climate events, and with more people moving into vulnerable locations, the probability of disasters is expected to increase unless additional efforts are undertaken to reduce losses in health, livelihoods, and lives [3].

Paragraph 31 of the 2030 Agenda for Sustainable Development calls for *the widest possible international cooperation aimed at accelerating the reduction of global greenhouse gas emissions and addressing adaptation to the adverse impacts of climate change*. In December, the Paris Agreement reinforced that world governments have the political will to implement the Agenda. Every country pledged to reduce emissions of greenhouse gases, strengthen resilience to the risks associated with a changing climate, and act nationally and internationally to address climate change. Countries pledged to strengthen their ability to prepare for, cope with, respond to and recover from climate-related risks, even as they built less carbon-intensive, resilient futures.

Further, the World Health Organization (WHO), health and environmental health non-governmental organizations, and others are raising awareness of the health risks of climate change (e.g. http://www.who.int/mediacentre/commentaries/climate-change/en/). WHO held its first international conference on climate change and health in 2014, with nearly 400 delegates from all regions, and its second in 2016 with more than 300 delegates invited. Senior government officials, leading scientists, health practitioners, and development partners agreed unanimously that climate change poses unacceptable risks to global public health, today and in the future. This is increasing awareness that focusing communication on the health risks of climate change is an effective approach to building public consensus on adaptation and mitigation [4].

However, as we discuss, health research institutes and organizations have not matched national and international political commitments with investments in scientific research into understanding, managing, and monitoring the current and projected health risks of global change. Health research is not keeping pace with the needs of local to national decision-makers for insights needed for effective policies and programs to protect population health and well-being today and in a very different future.

We briefly review the level of commitment of health research institutions and organizations to support research on climate change and health in the United States and Europe. We then discuss possible reasons for the disconnect between national political ambitions of importance for climate change and health and the funding support by those agencies with the mandate and budget to conduct the necessary research. We conclude with some options for moving to risk-centric, systems approaches that can provide useful and useable research to inform policies to increase the resilience of future populations.

## Commitment of health institutions and organizations to research on global change and health

In the United States, a 2009 review estimated the extent of Federal funding for research on the health risks of climate change was less than $3 million annually; this was funding across the National Institutes of Health (NIH), the Centers for Disease Control and Prevention; and the Environmental Protection Agency [5]. The review recommended intramural and extramural funding of more than $200 million annually to help the US prepare for, manage, and recover from the health risks posed by climate change. A separate review of the 2008 budget appropriations found that of the nearly 53,000 awards by NIH that year, approximately 0.17 % were focused on or related to climate [6]. The National Institute of Environmental Health Sciences (NIEHS), an institute within the National Institutes of Health, announced in 2011 that it would launch a research program in climate change and health. Its Strategic Plan 2012–2017 mentions climate change twice; one mentions climate change as a driver of changes in environmental exposures that could contribute to the worldwide increase in chronic, non-communicable diseases. The second mention is as an example in Goal 5 to focus on research to help inform policy responses. The annual budget reported by NIH to the US Global Change Research Program on climate change has been flat at $8 million for the past 4 years, or 0.025 % of the overall NIH budget of US$32 billion in 2016 and 0.3 % of the research budget of the US Global Change Research Program that coordinates federal research across agencies conducting research on climate change (budget is $2489 million [7]). A search of the NIEHS website lists 13 projects funded a few years ago that focused on some aspect of climate change and health [8]. There are no current solicited funding opportunities. In comparison, research on antibiotic resistant bacteria will receive $774 million in 2016. The President’s Executive Order, issued in November 2013, to prepare the US for the impacts of climate change, was an opportunity for NIH to consider the extent to which their research portfolio effectively addresses the health risks of climate variability and change in the US [9].

More than 70 multidisciplinary pan-European research projects addressing environment and health issues were funded by the European Commission’s Research and Innovation Directorate-General in the Seventh Framework Programme (FP7) of the European Union for Research, Technological Development, and Demonstration Activities (2007–2013). Of these, 11 projects focused on climate change and health with a budget of 41 million euros, or 0.08 % of the FP7 budget. The European Commission Third Health Programme 2014–2020 (Horizon 2020) mentions climate change as an example of a cross border threat where research is needed on implementation of European Union legislation on communicable diseases and other health threats [10]. Of the Horizon 2020 projects, six address climate change and health with a budget of 31 million euros; this is 0.04 % of the Horizon 2020 budget (http://cordis.europa.eu/projects/result_en?q=contenttype%3D'project'%20AND%20'Climate'%20AND%20'Change'%20AND%20programme/pga%3D'H2020*). Projects with less than 5 % of the estimated budget dedicated to health were not included.

Among major medical foundations, the Wellcome Trust recently began funding research on global change and health; it will invest GBP75 million over the next 5 years in projects focusing on urbanization and health, and global food systems and health [11]. Other major health foundations express concerns about the health risks of climate change, but their websites do not list programmatic funding.

The mismatch between acknowledgement of the health risks posed by climate change by major health research institutes and organizations and funding priorities is not only due to limited research funds in general, but likely due to multiple issues and competing priorities to better manage current health burdens. Two issues may contribute to this mismatch: viewing climate change as primarily an environmental problem, and therefore the responsibility of other research streams; and narrowly framing the health risks of climate change from the perspective of medicine and traditional public health, not from a systems-based approach considering the multiple interacting socioeconomic and environmental drivers.

## Climate change is not only an environmental problem

Climatologists and atmospheric chemists first raised concerns about climate change, specifically how increasing atmospheric concentrations of carbon dioxide from continued emissions of greenhouse gases and deforestation could impact global mean surface temperature, and the potential consequences of changing temperatures for natural systems such as agriculture and water resources [12]. This squarely framed climate change as an environmental, not a public health, concern. This perspective persists to this day, despite evidence of the impacts of a changing climate to population health and human well-being [13]. Within climate change research, assessment, and practice, it is now well recognized that the consequences of climate change can be pervasive and have repercussions throughout many facets of society and public health, and will likely compound existing or create new health inequities [13]. This understanding and its implications for public health, however, are not widely understood by public health decision-makers, researchers, and practitioners. Both low awareness of risks, and how these risks are conceptualized, affect actions taken today and in the future by individuals and health systems to manage the risks, and will affect the urgency associated with those actions.

Further, climate change will affect not just the burden of climate-sensitive health outcomes, but also will affect the ability of health systems to deliver critical services, such as managing supply chains during and after an extreme weather and climate event. These larger risks for the functioning of health systems have received less attention than projecting changes in disease burdens.

Climate change will not affect public health in isolation. The extent to which the current and future magnitude and pattern of changes in weather patterns may have adverse consequences for human health and well-being will depend on changes in other drivers of population health. Over the short term, these other changes are likely to be more important than climate change itself [13]. Development choices will determine the magnitude and pattern of vulnerability of future populations and regions [14]. Therefore, managing the health risks of climate change requires partnership in research and in practice outside the traditional boundaries of research domains and of ministries of health and schools of public health. Whether climate change turns out to be a major public health and health care challenge by mid-century and beyond depends on understanding the population health consequences of changes in human and natural systems and in development pathways.

## Relearning lessons learned

As a generalization, the history of environmental health is a series of ignored warnings of adverse health consequences from exposure to a particular agent, followed by an event with significant morbidity and/or mortality, before investment in research and development to prevent exposure and manage the consequences. Climate change is on the same trajectory. For more than 20 years, there have been warnings of the potential consequences of climate change on a wide range of health outcomes [15]. For example, climate and health researchers have been warning that warming temperatures could increase the geographic range and incidence of vectorborne diseases. Yet now that the Americas are struggling with large outbreaks of Zika virus, calls for research are focusing on vaccine development and not also considering environmental and social factors (e.g. travel) that can be used to forecast hotspots of transmission [16, 17].

## Mental models and frameworks: useful heuristics and possible straightjackets

Public health has long grappled with questions of causation and how to know whether an agent affects human health. Epidemiology was founded based on concerns of infectious diseases, leading to mental models applied to this day. Epidemiology has undoubtedly advanced human health and well-being with discoveries and understanding that saved the lives of countless millions. The typical reductionist approaches used in epidemiology are effective for solving certain problems, such as the health impacts of infection with a particular pathogen. Further, using a heuristic approach of acting in the absence of complete evidence through trial-and-error has served public health well. However, the underlying framing also creates straightjackets that can limit thinking about wicked problems such as climate change. There appears to be widespread confusion of necessary vs. sufficient causes of adverse health outcomes. While it is necessary for the Zika virus to be present for it to be transmitted by *Aedes* mosquitoes, virus presence is not sufficient for disease transmission [18]. Understanding the system within which disease transmission occurs requires knowing relationships among climatic, environmental, social, economic, health, and other drivers of disease occurrence [19]. Limited health research aims at systems-based understanding of risks and effective responses.

Since the 1980s, toxicological risk assessment has been the prevailing framework in public health for assessing the possible health impacts of environmental exposures (e.g. [20]). Although risk assessment continues to be extremely useful at identifying potentially hazardous environmental and occupational exposures and the concentrations that are likely to be safe for susceptible human populations, this approach was not designed for understanding and quantifying multiple, interacting factors that change over time and, therefore, has limited applicability for understanding the health risks of climate change [21]. Climate change is associated with changes in the mean and variability of weather variables, sea level rise, and ocean acidification, which then affect environmental conditions and ecosystems that are determinants of the magnitude and pattern of the burden of climate-sensitive health outcomes. Exposure-response endpoints vary from location to location because of the local context, and will vary over time as exposure and vulnerability continue to change. In general, the risk that a particular weather pattern will present to a population will depend not just on the exposure, but also on the population sensitivity to that exposure and the ability of public health and health care organizations to prepare for and cope with the exposure and its consequences [20, 22]. Therefore, understanding the risks of climate change requires understanding the local and regional context within which impacts could occur. Vulnerabilities, such as poverty or failing infrastructure, may be more important determinants of the magnitude and pattern of climate-sensitive health outcomes over the next few decades than changes in weather patterns [13]. Incorporating the local context, including local stakeholders, is critical for effectively identifying and managing risks [23]. Further, risk assessment also does not answer critical questions on prioritizing interventions across a wide range of climate-sensitive health outcomes, or on their effectiveness. A modified evidence-based public health approach could more effectively accommodate the wide-ranging exposures, outcomes, and modes of inquiry associated with climate change and health adaptation [24].

As discussed by McMichael [15], the traditional epidemiologic focus on proximate, individual level risk factors is insufficient to understand and manage the health risks of global change. Other frameworks for approaching complex risks include eco-social [25] and ecohealth, which focuses on how changes in the earth’s ecosystems can affect human health [26]. These frameworks facilitate understanding of the complex interactions and dependencies across environmental, social, and human systems that determine risk. They aid in understanding that the relationship between exposure and adverse health outcome can be affected by many other factors. For example, socioeconomic factors play a critical role in altering vulnerability and sensitivity, by interacting with biological factors that mediate risk and/or lead to differences in the ability to manage stressors.

Instead, the climate change risk and adaptation communities focus on risk and risk management. The risks of climate change are a function of the hazards that can arise with climate change; the individuals and communities exposed to those hazards; and their associated susceptibility to harm and ability to cope, respond, and recover [20]. Focal areas of research within this framing include understanding vulnerabilities to current impacts and future risks, identifying populations and regions expected to have higher levels of exposure, the expected consequences of exposures and vulnerabilities within the context of different climate and development pathways, effective approaches to inform policies and measures to ameliorate current problems and projected challenges, and decision-making processes themselves (e.g. [20]). The methods, inputs, and outcomes from a standard risk assessment as applied in the health sector, explicitly incorporates stakeholders, consideration of the local context, and systems-based approaches. These vulnerability and adaptation assessments are being conducted worldwide, with lessons learned on how to increase their effectiveness in the context of health systems [27, 28]. An example is the U.S. Centers for Disease Control and Prevention framework on Building Resilience Against Climate Effects (BRACE) [29]. This five-step process helps state and local health officials in the U.S. anticipate, prepare for, and respond to the health risks of climate change by projecting future health burdens with climate change, assessing interventions to reduce the risks, developing a climate and health adaptation plan, and evaluating the process and improving implemented options.

## Merging top-down and bottom-up public health

Public health successes since establishing public health institutions (in the west) in the 1840s have primarily been top-down, with a health risk identified (e.g. cholera) and then preventive measures developed and deployed uniformly (e.g. improving sanitation systems and, eventually, vaccination). Public health has had a remarkable track record with these single-issue, vertical programs in reducing the disease burden in susceptible populations by targeting the proximal causes of disease, such as infectious pathogens, physical activity, nutrition, or tobacco and alcohol use, that can be controlled at the individual level [15, 30]. Climate change presents an opportunity to reconfigure top-down approaches to incorporate how local socio-environmental factors can increase or decrease vulnerability to particular hazards, of how those factors can be most effectively managed, and of how the sensitivity of exposed populations and the capacity of health systems to prepare for and manage risks will affect the success of health policies and measures. This requires new approaches to assessing risks, formulating interventions, and conducting monitoring, evaluation, and learning to increase the efficiency and effectiveness of health systems in protecting population health.

Rather than solely focusing on individual-level interventions, contextual interventions are needed that target the environmental and socioeconomic setting within which interventions are implemented. Adaptation, ecohealth, eco-social, and other frameworks promote integrating top-down and bottom-up approaches [25, 26, 31] One public health strategy designed to enhance community resilience to climate change is lateral public health [32]. This approach is based on the understanding that community-based adaptation can build on collective capacity inherent in social networks to advance resilience to climate change and other factors. Lack of social networks and limited social support may predispose urban residents to adverse health outcomes in extreme situations. Conversely, robust social networks are important pillars for positive health outcomes during climate-related events such as heatwaves. For example, participating in group activities such as clubs, support groups, churches, etc., or having a friend close by, is protective against heat-related mortality [33]. Conversely, those who live alone or who do not have a pet to care for are at increased risk of succumbing to heat weather. Lateral public health empowers officials, agencies, and local communities to collaborate and to seek solutions to their challenges. A number of community interventions have been implemented by community members such as building green roofs and urban gardens to mitigate the heat island effect, bio-swales for urban runoff, etc. [34, 35]. By engaging different actors, lateral public health helps with mainstreaming climate change adaptation into a range of other programs and sectors with a number of co-benefits. Public health practitioners can mount an effective adaptation response to climate change if they reach out to all stakeholders, including those not traditionally associated with their discipline. Thus, lateral public health is based on inter-sectorial cooperation and community-based participation [32]. It is grounded in community-based participatory research, volunteerism, and integrated public health practice, and has the potential to improve linking social capital by connecting community members with city administration. Because public health funding for climate change adaptation is limited, a lateral public health strategy offers an approach to meet some of future challenges by relying on skills and capacity within the community. This, however valuable, will be insufficient to address risks that health systems should be providing, such as building early warning and response systems or projecting risks over coming decades to inform prioritization of adaptation and mitigation options.

## Protecting population health over coming decades requires investment in gathering evidence of and projecting risks, and in identifying additional interventions to protect health and health systems

Shifting priorities and increasing the limited research funding are critical because impacts of climate change are already evident, with impacts projected to increase over coming decades [13]. Health policy- and decision-makers don’t have the information needed to prepare for and manage those risks. Research is needed not just on the magnitude and pattern of current risks, but also how risks could evolve this century under different assumptions about climate and development. Health determinants and drivers will change over time; their respective contribution to future health burden needs to be assessed. Evidence and modeling can be used to identify the most important determinants of climate-sensitive health outcomes, and their contribution to future health burden incorporated into projections of the magnitude and pattern of likely future risks. This requires models and scenarios to provide insights into where and when risks could arise, and the circumstances under which various interventions (adaptation and mitigation) could reduce risks over spatial and temporal scales [14, 36]. Models are needed not just of specific health risks, but also models that integrate across sectors, such as how changes in climate could affect water, agriculture, and, ultimately, undernutrition and micronutrient deficiencies.

Besides an increase in the commitment of health institutions and organizations to supporting health research on global environmental change, a shift in priorities is also called for. Most importantly, government-funded research should, in part, be informed not just by scientific research gaps and needs, but also by environmental justice concerns. Such priorities should also clearly reflect the priorities of the 2030 Agenda for Sustainable Development, the Sendai Framework for Disaster Risk Reduction, and the Paris Agreement under the United Nations Framework Convention on Climate Change (Table 1). Research also should help identify best practices in ensuring effective functioning of health systems in a changing climate.

The major national and international governmental organizations funding health research should consider these priorities and tailor calls for research proposals towards interdisciplinary teams that transcend the traditional disciplinary divide. Research projects should also be coupled with community-based interventions to assure the translation of research findings into practice. Implementation of viable intervention studies that can be replicated in other settings is desirable. Funding agencies should therefore require that community partners join research consortia in order to help shape the research question, study design, implementation of the intervention and evaluation. Needless to say, this approach would have far-reaching institutional consequences, including extension of project duration, funding cycles, and evaluation processes. Within academic settings, interdisciplinary research needs not just higher prioritization, but criteria for promotions need to reflect the value interdisciplinary teaching, research, and publications in preparing the next generation of an informed citizenry.

Further, health system policies are just beginning to be revised to explicitly incorporate climate variability and change. Understanding is needed of how best to modify current and implement new policies within the context of iterative risk management, so that resources are deployed efficiently, focusing on protecting individuals and communities from the current impacts of climate change while increasing the range of options available to future decision-makers to manage changing risks [37, 38].

## Conclusions

Repeated calls for moving from medical models of disease causation to frameworks that embrace and encourage systems thinking, integrating complex determinants of human health, including environmental, social, and biological drivers and well-being, have led to minor changes in funding priorities for climate and other global environmental changes [15, 25, 39]. Climate change is altering all aspects of life, with risks projected to increase substantially over this century even with proactive adaptation [13]. Effectively and efficiently preparing for and managing these risks requires significant investments in research and technology development that prioritize the needs of particularly vulnerable communities and locations, and that explicitly support the major commitments of national governments to undertaking actions to promote societal resilience. This is an opportunity for health organizations and institutions to contribute to broader societal goals to transition to sustainable development. The methods and tools are available, as is a growing base of researchers and practitioners. Taking that step requires medical research funding agencies to acknowledge and fund the research and development that will help individuals and societies prepare for a future that will differ in many aspects from today.

As Winslow stated in 1920:

*We need organizers and propagandists for the cause of health, capable of building wisely the great scheme of health protection of the future and of enlisting in its support the enthusiastic cooperation of the peoples of the earth* [40]*.*

## Abbreviations

BRACE: Building Resilience Against Climate Effects
FP7: European Commission’s Research and Innovation Directorate-General in the Seventh Framework Programme
NIEHS: United States National Institute of Environmental Health Sciences
NIH: United States National Institutes of Health
WHO: World Health Organization
